# Event and Comment

**Published:** 1916-08

**Authors:** 


### DENTAL REGISTER

Vol. LXX AUGUST, 1916 No. 8

### EVENT AND COMMENT

THE MODERN FIELD OF PRACTICE. The scope of dentistry at the present time is so comprehensive that few dentists are attempting to cover it in practice. We are rapidly dividing it into special lines of effort, and curiously each one seems to be large enough to satisfy the ambitious of the more aggressive workers. An eminent authority in one of our dental specialties recently asked us if it was not time to drop a general education as a professional dentist and train men for but one distinct line of practice. The necessity for more workers in the field of oral hygiene has compelled us to undertake the training of workers in this field entirely independent of the general practitioner. All this is indicative of the widespread belief that the care of the teeth is a most important health duty, and also of another important fact, namely, that technical dentistry has become so intricate and exacting that no one person is able to administer it adequately to any considerable number of people. At the present time it seems to be splitting up into three fairly well defined departments. First there is what may be called the relief work, which may include all forms of emergency effort to relieve suffering and rescue the threatened loss of function of the teeth as a whole or as individuals. This may be summarized as general or traditional practice. There will, of course, always be a demand for this form of dentistry, and in fact, unless something is done to check the disease processes in the mouth, we shall need to increase materially our facilities for training dentists to take care of this department alone. Then there is another closely allied department which includes various efforts to restore the functions of lost teeth. This may include all prosthetic efforts, such as crown and bridge and plate work. Instead of the demand in this department lessening because of modern hygienic efforts, there is an alarming increase in all the departments of prosthesis. The third department, which, though not new, is just now receiving unusual notoriety is preventive dentistry. This work is at present being studied and worked along the lines of oral hygiene and prophylaxis. This is in our judgment the most important line of work in the entire field, for the reason that it promises to be the surest way of controlling the continual increase in the other departments. At present the idea seems to take only the form of prophylactic care of children's teeth of school age. In our judgment we shall need to start further back and begin with the more elemental structures of tooth and jaw development. This means that we shall ultimately be forced to associate our efforts with our medical confreres and give more serious attention than has heretofore been customary to the developmental processes. We must have not only perfect teeth in properly formed jaws, but we must know what to eat and how to eat it. Its a big view and a long look ahead. Are we equal to it?

